# 559 Rates of Follow up in Massively Burn Injured Patients

**DOI:** 10.1093/jbcr/irae036.193

**Published:** 2024-04-17

**Authors:** Jason Heard, Jessica Valdez, Tina L Palmieri, Kathleen S Romanowski, Soman Sen, David G Greenhalgh

**Affiliations:** University of California - Davis Hosp, Sacramento, CA; UC Davis, Sacramento, CA; UC Davis, Shriners Children's Northern California, Sacramento, CA; Shriners Hospital Northern California, Sacramento, CA; University of California - Davis Hosp, Sacramento, CA; UC Davis, Sacramento, CA; UC Davis, Shriners Children's Northern California, Sacramento, CA; Shriners Hospital Northern California, Sacramento, CA; University of California - Davis Hosp, Sacramento, CA; UC Davis, Sacramento, CA; UC Davis, Shriners Children's Northern California, Sacramento, CA; Shriners Hospital Northern California, Sacramento, CA; University of California - Davis Hosp, Sacramento, CA; UC Davis, Sacramento, CA; UC Davis, Shriners Children's Northern California, Sacramento, CA; Shriners Hospital Northern California, Sacramento, CA; University of California - Davis Hosp, Sacramento, CA; UC Davis, Sacramento, CA; UC Davis, Shriners Children's Northern California, Sacramento, CA; Shriners Hospital Northern California, Sacramento, CA; University of California - Davis Hosp, Sacramento, CA; UC Davis, Sacramento, CA; UC Davis, Shriners Children's Northern California, Sacramento, CA; Shriners Hospital Northern California, Sacramento, CA

## Abstract

**Introduction:**

As burn care continues to improve, large total body surface area (TBSA) burn survival will increase. These survivors require more extensive care than smaller burns and are at higher risk for wound/scar related complications. In previous studies follow up after burn injuries tended to be low, especially with burns that cover a smaller TBSA%. The low follow up compliance has been linked to socioeconomic factors such as housing insecurity and substance use. There are limited studies that evaluate socioeconomic factors that contribute to follow up attrition in massive burn patient populations We hypothesize that patients with massive burn injuries and housing insecurity and substance use will have lower rates of follow up.

**Methods:**

After approval by our Institutional Review Board, a retrospective chart review study was performed in adult patients admitted from 2009-2019 with >50% TBSA burns. Patients were excluded if they died during hospitalization or were discharged with plan to follow up at another burn center. Data collected included demographics, injury characteristics, zip codes, insurance type/status, follow up visits and number/type of reconstructive surgeries. County level data was abstracted from data.census.gov which. Univariate and regression analyses were performed using R software.

**Results:**

There were 155 patients that met the inclusion criteria, 72 patients survived to discharge, and 7 patients were discharged to another burn center leaving 65 patients in the final analysis. Mean TBSA of all patients was 63.5% and mean age was 35.6 years. Fifty-four patients (83.1%) attended at least one follow up after burn injury. The median number of follows up scheduled and attended were 4 and 3.5 (range: 1-135) respectively. On Univariate analysis female patients followed up longer than male patients (771 vs 131 days; p=0.007) and patients with housing insecurity were less likely to follow up at all (4 vs 0 visits; p=0.0009). On multivariate regression analysis for attending any clinic visit, larger TBSA burn was independently associated with follow up (OR 1.22, p=0.046) and housing insecurity was independently associated with not following up (OR 0.003, p=0.02).

**Conclusions:**

Patients with large TBSA% burn injuries are a unique patient population and in theory should require more frequent follow up than patients with smaller TBSA burns. Data from our single center study showed patients that survived massive burn injuries did have reasonable one time follow up. However, certain patient populations should be targeted for closer follow up adherence, including patients facing housing insecurity, uninsured patients, and male patients.

**Applicability of Research to Practice:**

This research can help other burn programs identify patients who may be vulnerable to have higher follow up attrition rates so that these specific patient populations can be followed closely after discharge.

